# Increased ROS production and DNA damage in monocytes are biomarkers of aging and atherosclerosis

**DOI:** 10.1186/s40659-018-0182-7

**Published:** 2018-09-05

**Authors:** Thais A. Jacinto, Giselle S. Meireles, Ananda T. Dias, Rafaela Aires, Marcella L. Porto, Agata L. Gava, Elisardo C. Vasquez, Thiago Melo C. Pereira, Bianca P. Campagnaro, Silvana S. Meyrelles

**Affiliations:** 1 Laboratory of Translational Physiology, Health Sciences Center, Federal University of Espirito Santo (UFES), Vitoria, Brazil; 2 Laboratory of Translational Physiology and Pharmacology, Pharmaceutical Sciences Graduate Program, Vila Velha University (UVV), Rua Mercúrio, s/n, Boa Vista 1, Vila Velha, ES 29102-623 Brazil; 3 Federal Institute of Education, Science and Technology (IFES), Vila Velha, ES Brazil; 4 Division of Nephrology, McMaster University, Hamilton, ON Canada

**Keywords:** apoE knockout mice, Atherosclerosis, Proinflammatory cytokines, Apoptosis

## Abstract

**Background:**

New evidence demonstrates that aging and dyslipidemia are closely associated with oxidative stress, DNA damage and apoptosis in some cells and extravascular tissues. However, in monocytes, which are naturally involved in progression and/or resolution of plaque in atherosclerosis, this concurrence has not yet been fully investigated. In this study, we evaluated the influence of aging and hypercholesterolemia on serum pro-inflammatory cytokines, oxidative stress, DNA damage and apoptosis in monocytes from apolipoprotein E-deficient (apoE^−/−^) mice compared with age-matched wild-type C57BL/6 (WT) mice. Experiments were performed in young (2-months) and in old (18-months) male wild-type (WT) and apoE^−/−^ mice.

**Results:**

Besides the expected differences in serum lipid profile and plaque formation, we observed that atherosclerotic mice exhibited a significant increase in monocytosis and in serum levels of pro-inflammatory cytokines compared to WT mice. Moreover, it was observed that the overproduction of ROS, led to an increased DNA fragmentation and, consequently, apoptosis in monocytes from normocholesterolemic old mice, which was aggravated in age-matched atherosclerotic mice.

**Conclusions:**

In this study, we demonstrate that a pro-inflammatory systemic status is associated with an impairment of functionality of monocytes during aging and that these parameters are fundamental extra-arterial contributors to the aggravation of atherosclerosis. The present data open new avenues for the development of future strategies with the purpose of treating atherosclerosis.

## Background

Despite improvements in pharmacological and non-pharmacological interventions, atherosclerosis is still the leading cause of death in western countries [1, 2] and considered an epidemic disease worldwide [3]. In the last decade, many studies have provided an extraordinary progress in the understanding of this disease, and cumulative evidence shows that lipoprotein metabolism disorders per se cannot sufficiently explain the development of atherosclerotic lesions [4]. Additional factors such as aging, oxidative stress, endothelial dysfunction and activation of the immune system have also been considered important modulators for the aggravation of the atherosclerotic disease [5–8]. Another important open question in this process is the involvement of key extravascular cells related with inflammation, such as the monocytes.

In conditions of hypercholesterolemia, monocytes migrate from the blood stream targeting injured vascular tissues, where these cells differentiate into macrophages [9–11]. Monocyte-derived macrophages uptake oxidized low-density lipoprotein (ox-LDL) and become lipid-rich foam cells, leading to the release of pro-inflammatory cytokines which in turn contributes to formation of atheroma [11]. Sequentially, into the plaques, the cholesterol crystals inhibit the macrophage autophagy process, exacerbating the inflammatory response and contributing to the defective efferocytosis [12]. This process results in the formation of unstable plaques [12].

Considering the close and dynamic contact of peripheral blood cells with the arterial wall [13, 14], an amplified systemic inflammation response is a major factor contributing to the aggravation of atherosclerosis and could predict future adverse clinical outcomes.

We have recently demonstrated in the apoE^−/−^ mouse model of spontaneous atherosclerosis, that the concurrence of aging and hypercholesterolemia is associated with oxidative stress and DNA damage in undifferentiated bone marrow cells, which are very important for tissue maintenance and repair/regeneration [15]. Therefore, it is important to consider that peripheral blood cells play an important role in atherogenesis promoting and perpetuating the recruitment of bone marrow monocytes [16–19].

Although many studies have been conducted to evaluate various mechanisms involved in atherosclerotic disease, the effects of increased production of reactive oxygen species (ROS) in monocytes of hypercholesterolemic and aged mice has not yet been completely elucidated. The aim of the present study was to test the hypothesis that the concurrence of hypercholesterolemia and aging lead to enhanced production of oxidative stress and DNA damage in systemic monocytes.

## Results

### Blood cells counting

**Fig. 1:**
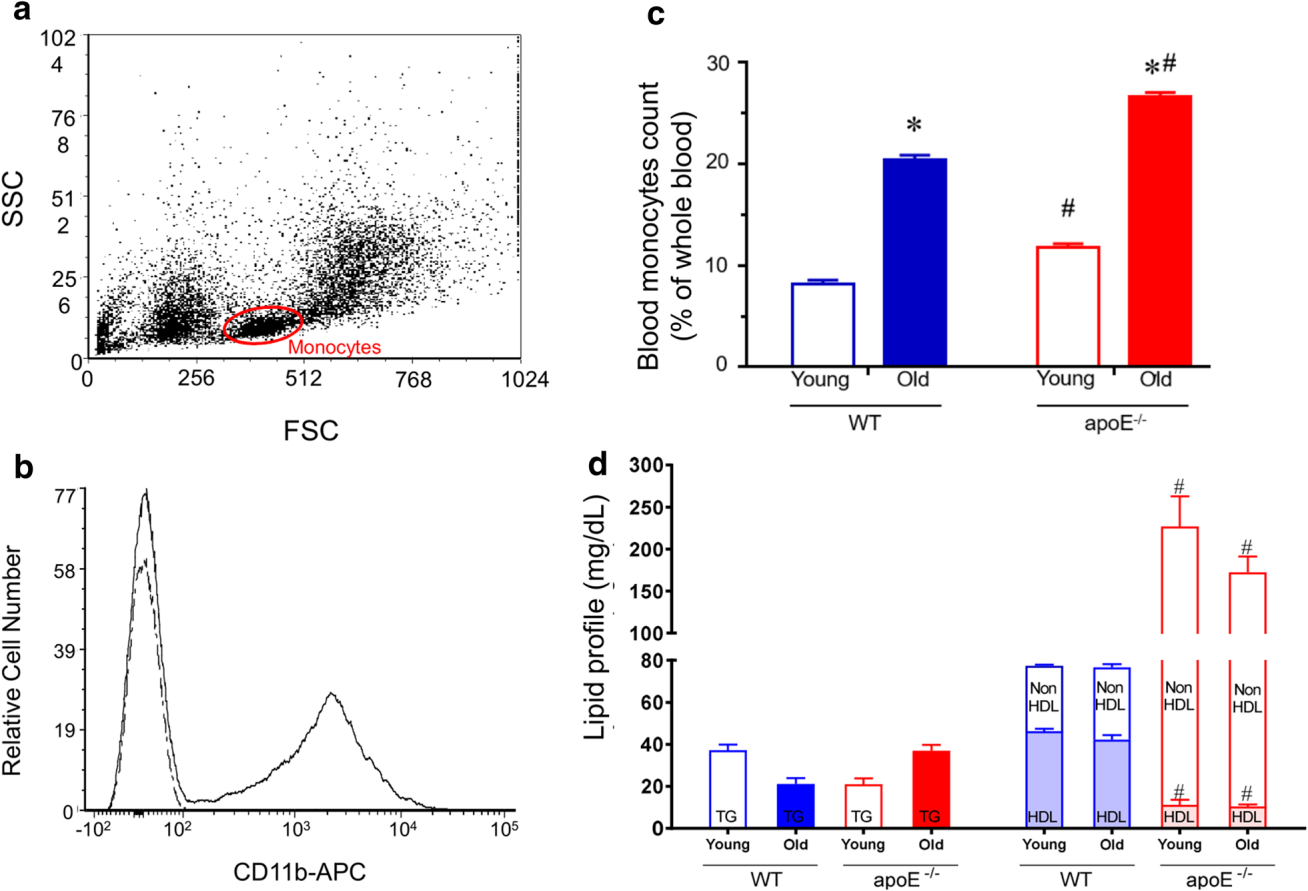
**a** The FSC scatter data provide information on the relative size of the cells, whereas the SSC data estimate the granularity. Dot plot where each dot represents one event. Here only 10,000 events are shown to avoid oversaturation of dots. Monocyte population defined by FSC and SSC properties are shown on the graph. **b** Peripheral blood cells were stained with anti-CD11b-APC antibody or isotype control to confirm the monocyte population. The flow cytometric immunophenotipic data revealed that the subset of monocytes defined by FSC and SSC expressed CD11b antigen (~ 40% positive cells). **c** Monocytes as percentage of whole lysed (depleted erythrocytes) peripheral blood cells. Bars graph represents the proportion of total circulating monocytes in apoE^−/−^ and age-matched WT mice. **d** Lipid profile in apoE^−/−^ and WT mice. Values are means ± SEM, *p < 0.05 vs. the respective young group, ^#^*p *< 0.05 vs. age-matched WT group

Considering that aging and atherosclerosis could influence the blood cell populations, we used flow cytometry to determine the percentage of circulating monocytes in blood. The percentage of monocytes in the apoE^−/−^ mice was significantly augmented in both young (11.8 ± 0.3%) and old (26.6 ± 0.4%) mice compared with WT (8.2 ± 0.3 and 20.4 ± 0.4%, respectively) (Fig. 1c).

#### OGTT and biochemistry parameters

In order to evaluate other metabolic parameters that could be affected by aging and particularly by atherosclerosis, we measured glycemia and observed that the values were similar in young and aged WT (76 ± 4 vs. 90 ± 5 mg/dL, respectively) and apoE^−/−^ (96 ± 15 vs. 105 ± 10 mg/dL, respectively) mice. Regarding OGTT data, no significant difference was observed between groups in WT (young: 24786 ± 534 vs. old: 26727 ± 580 AUC_0–120_) and apoE^−/−^ (young: 24313 ± 2261 vs. old: 21928 ± 2741 AUC_0–120_).

Serum lipid profile is represented in Fig. 1d. As expected, all the apoE^−/−^ groups showed an increase in non-HDL cholesterol (young: 216 ± 36 and old: 162 ± 19 mg/dL, p < 0.05) compared with the WT mice (31 ± 1 and 34 ± 2 mg/dL, respectively). Regarding HDL, a reduction was observed (young: 11 ± 2 and old: 10 ± 1 mg/dL, p < 0.05) compared with the WT mice (46 ± 1 and 42 ± 2 mg/dL, respectively), without any change in triglycerides in all groups.

### Inflammation analyses

**Fig. 2:**
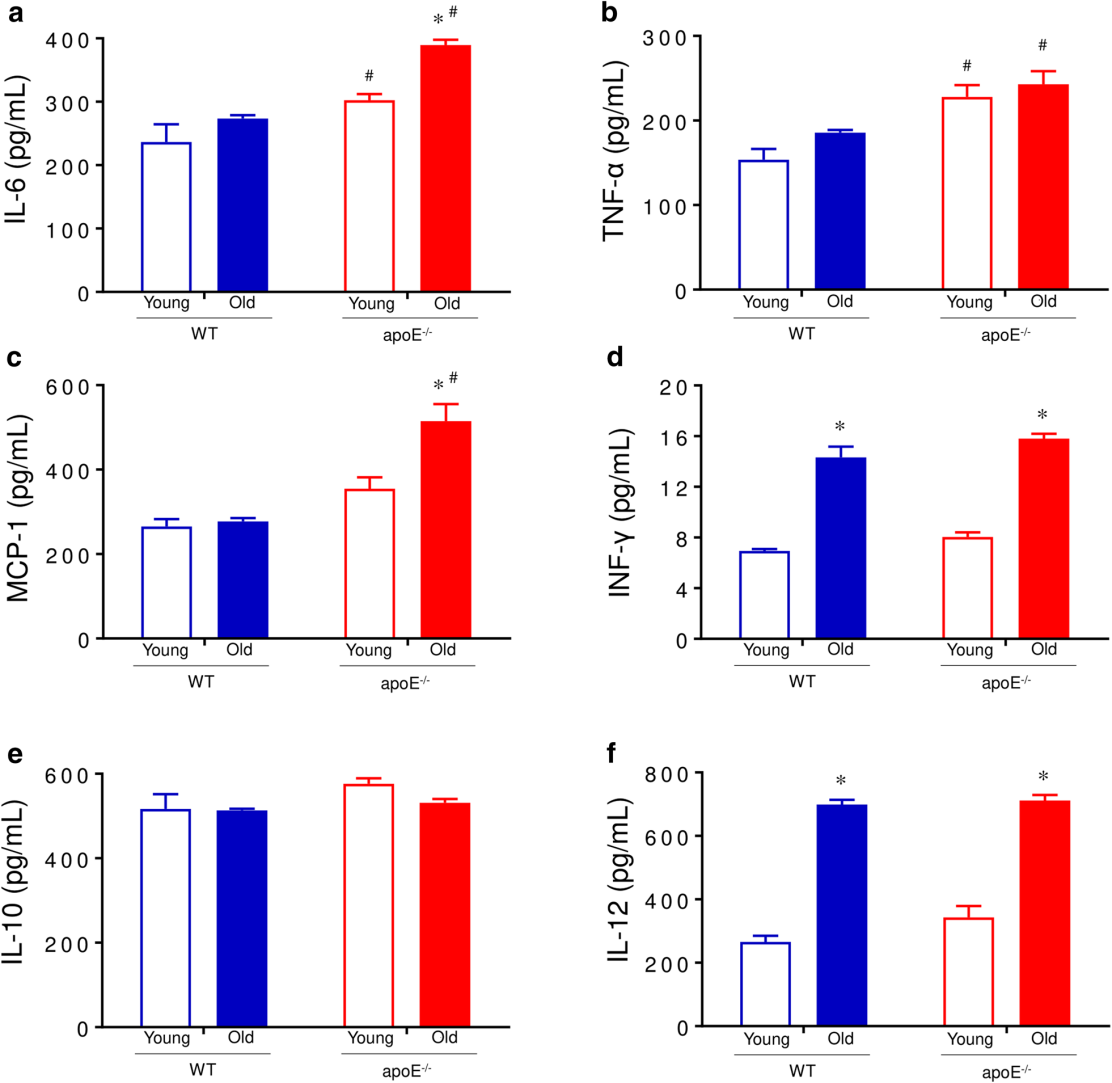
Serum cytokine profile. Protein levels of pro-inflammatory cytokines in apoE^−/−^ versus WT mice. Serum levels of IL-6 (**a**), TNFα (**b**), MCP-1 (**c**), INF-γ (**d**), IL-10 (**e**) and IL-12 (**f**) were evaluated by flow cytometry. Atherosclerotic aged mice showed augmented pro-inflammatory cytokine expression compared with WT. Values are means ± SEM, ^#^*p *< 0.05 vs. age-matched WT group

Six cytokines that are known to be involved in inflammation were quantified by flow cytometry with the CBA system. As shown in Fig. 2, aged atherosclerotic mice exhibited a significant increase in the levels of biomarkers of inflammation IL-6 (42%), TNF (30%) and MCP-1 (85%) compared to age-matched WT mice. Aging by itself caused a significant increase in the levels of IL-12 (WT: 2.6-fold; apoE^−/−^: twofold, p < 0.05) and INFγ (WT: 2.1-fold; apoE^−/−^: twofold, p < 0.05). No significant difference was observed in IL-10 levels between groups.

### ROS production

**Fig. 3:**
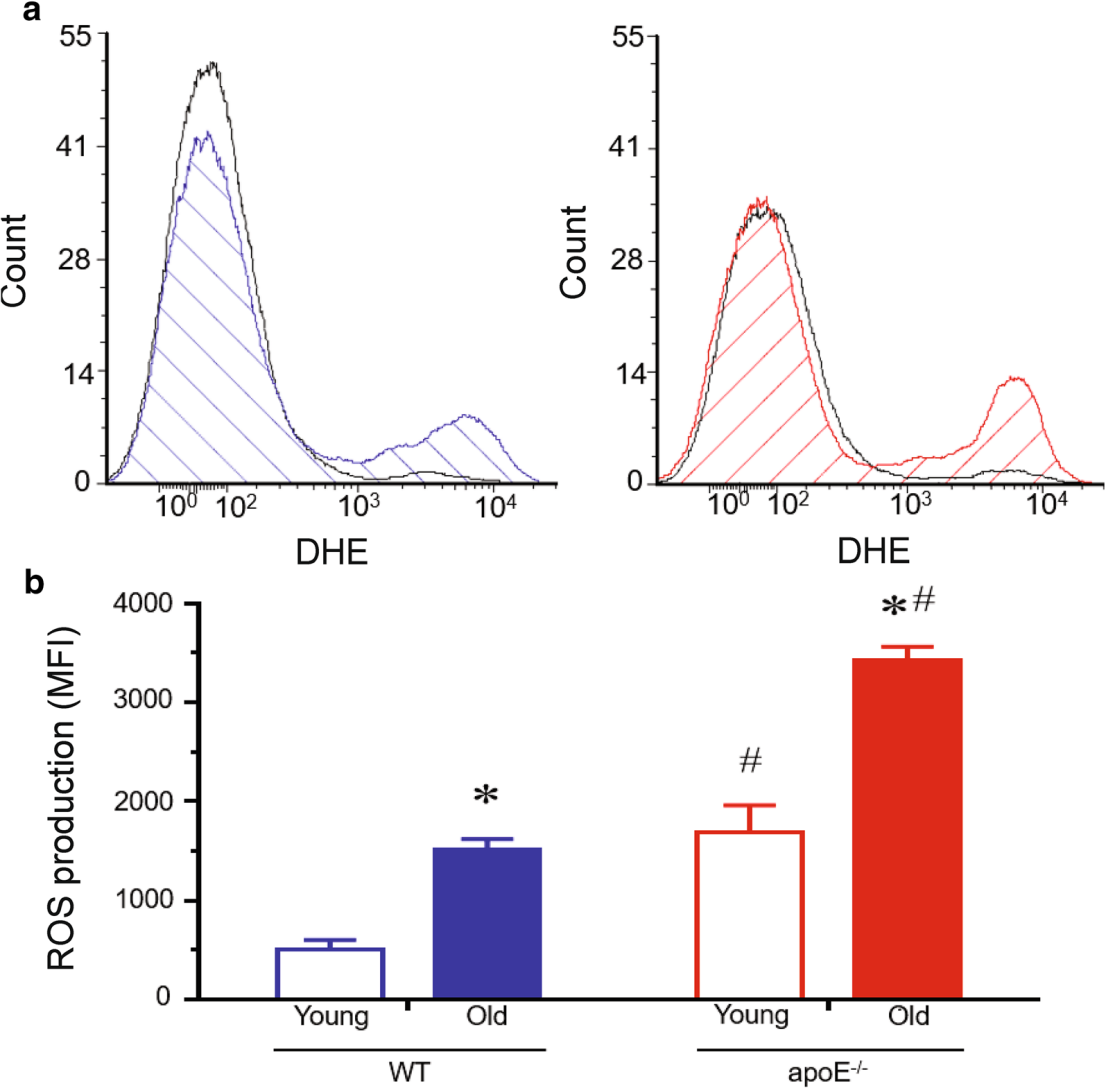
Increased systemic ROS production in apoE^−/−^ mice. **a** Top panel shows representative histogram with DHE staining of monocytes. **b** Bar graph shows the difference on MFI of DHE measured by flow cytometry in monocytes. Blue line: old-WT; red line: old-apoE^−/−^. Values are means ± SEM, *p < 0.05 vs. the respective young group, ^#^*p *< 0.05 vs. age-matched WT group

**Fig. 4:**
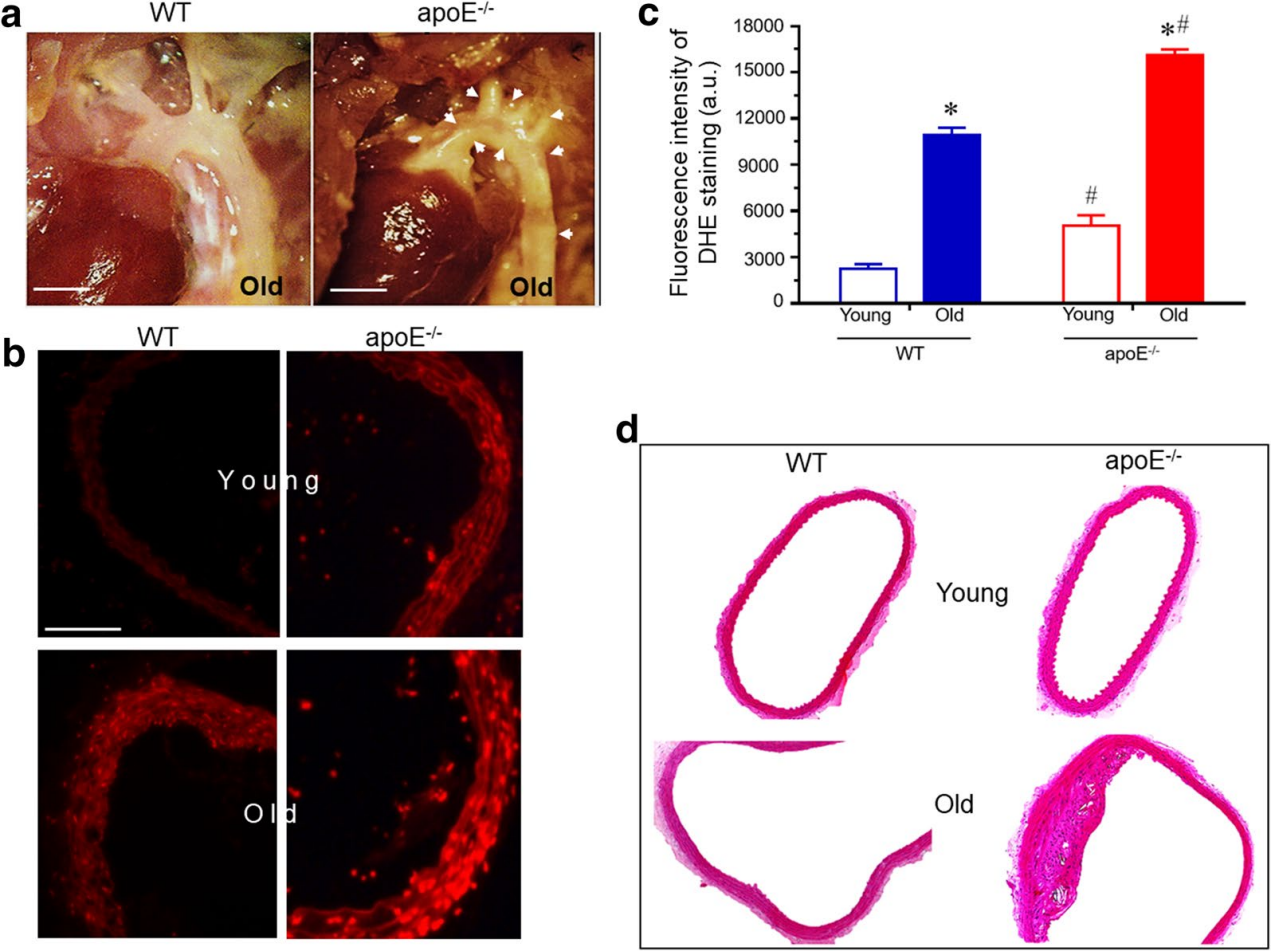
**a** Representative photographs of mice aortic arch anatomy in situ. Atherosclerotic plaque formation is notably greater in apoE^−/−^ mice, while no lesion was present in the age-matched WT control. Arrow heads indicates plaque deposition (4×; Bar = 2 mm). **b** DHE staining of representative aortic arch cross-sections. Atherosclerotic mice showed increased DHE-positive cells compared to WT (magnification 400×; Bar = 100 µm). **c** Quantification of ROS production. Aging increased ROS production in aortic arch cross-section and it was aggravated through hypercholesterolemia. **d** Representative micrographs of aortic arch stained with hematoxylin and eosin. Values are means ± SEM, **p < 0.01 vs. WT. *p < 0.05 vs. the respective young group, ^#^*p *< 0.05 vs. age-matched WT group

The production of ROS was determined by the DHE staining approach in both monocytes (Fig. 3) and in aorta sections (Fig. 4). Top panel of Fig. 3 shows a typical flow cytometry histogram of monocytes. The histogram showed a rightward shift in the log of DHE fluorescence in old compared to young WT (blue and black lines) and apoE^−/−^ (red and black lines) mice (Fig. 3a). As summarized in the bottom bar graph, old apoE^−/−^ mice exhibited a remarkable increase in ROS production (expressed in MFI) in monocytes compared with young apoE^−/−^ mice (3424 ± 129 vs. 1682 ± 277 a.u., p < 0.05, respectively) and compared to age-matched WT mice (1519 ± 98 vs. 508 ± 92 a.u., p < 0.05, respectively, Fig. 3b). Figure 4 panels show typical images from aorta and, as expected, old apoE^−/−^ mice exhibited notable lipid plaques which was not observed in old WT mice (Fig. 4a). Figure 4b and c shows the ROS production in aortic arch from apoE^−/−^ mice compared to WT. The typical microscopic images show an augmented DHE staining in the cross sections of aorta of apoE^−/−^ mice compared to age-matched WT (Fig. 4b). Bottom bar graph in Fig. 4c summarizes the analysis of ROS. The old hypercholesterolemic mice show a remarkable increase in ROS production in aortic arch (16097 ± 395 a.u., p < 0.05) compared with age-matched WT (10947 ± 459 a.u.) animals. Similarly, the aging process shows increased oxidative stress in mice’s aortic arch, when comparing old and young WT (2223 ± 327 a.u.) and apoE^−/−^ (5046 ± 670 a.u., p < 0.05) mice.

Figure 4d shows typical photomicrographs of aortic arch sections stained with hematoxylin and eosin. The image of the aortic arch from aged apoE^−/−^ mice shows a developed plaque area, while the image for young atherosclerotic and WT mice shows no structural differences.

### DNA content

**Fig. 5:**
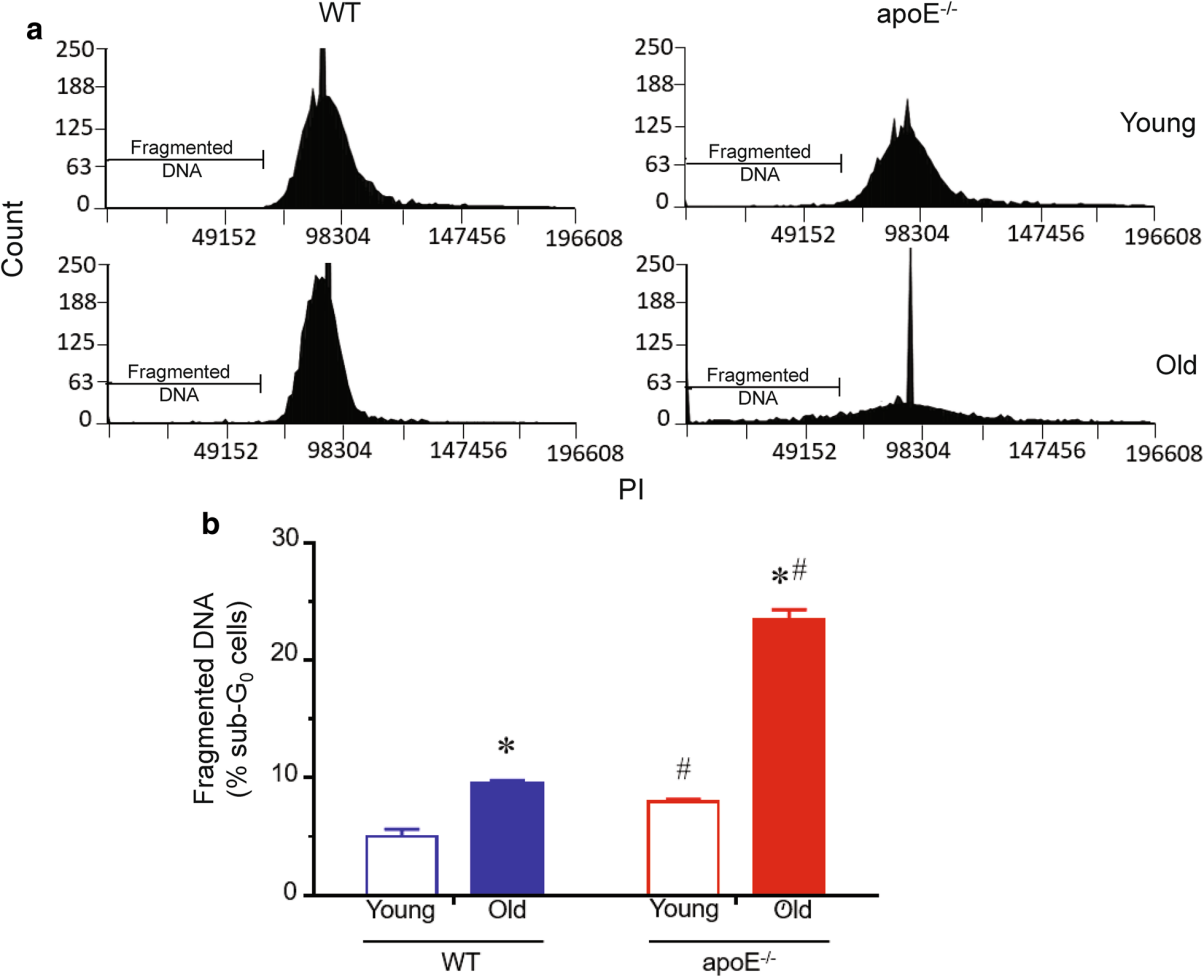
DNA content analysis. **a** Typical histograms of cell cycle distribution of monocytes from young and old WT and apoE^−/−^ mice. **b** Bar graph shows percentage of cells with fragmented DNA in monocytes. Values are mean ± SEM. *p < 0.05 vs. the respective young group, ^#^*p *< 0.05 vs. age-matched WT group

Given that hypercolesterolemia increases the ROS production in apoE^−/−^ mice, and that this stimulus can interact with and damage the DNA, we tested the DNA fragmentation by cell permeabilization followed by PI labeling (Fig. 5). As illustrated in the DNA histograms, apoE^−/−^ mice showed a subtle increase in fragmented DNA in monocytes (Fig. 5a) compared to age-matched WT mice. The total fragmented DNA is summarized in Fig. 5b, confirming the augmented DNA fragmentation in atherosclerotic mice (young: 8.0 ± 0.2; old: 23.0 ± 1.0%, p < 0.05) compared to WT (young: 5.0 ± 0.6; old: 9.5 ± 0.3%, p < 0.05) animals. In addition, aging per se was able to increase DNA damage in both WT and apoE^−/−^ (WT: 60%; apoE^−/−^: 142%, p < 0.01).

After the analysis of DNA damage, we decided to verify the statistical relationship between ROS production and DNA fragmentation. For this purpose, a correlation test was applied, which showed a significant positive correlation between these two variables in old and hypercholesterolemic animals (WT young: r = 0.65; WT old: r = 0.84; apoE^−/−^ young: r = 0.86; apoE^−/−^ old: r = 0.96).

### Apoptosis

**Fig. 6:**
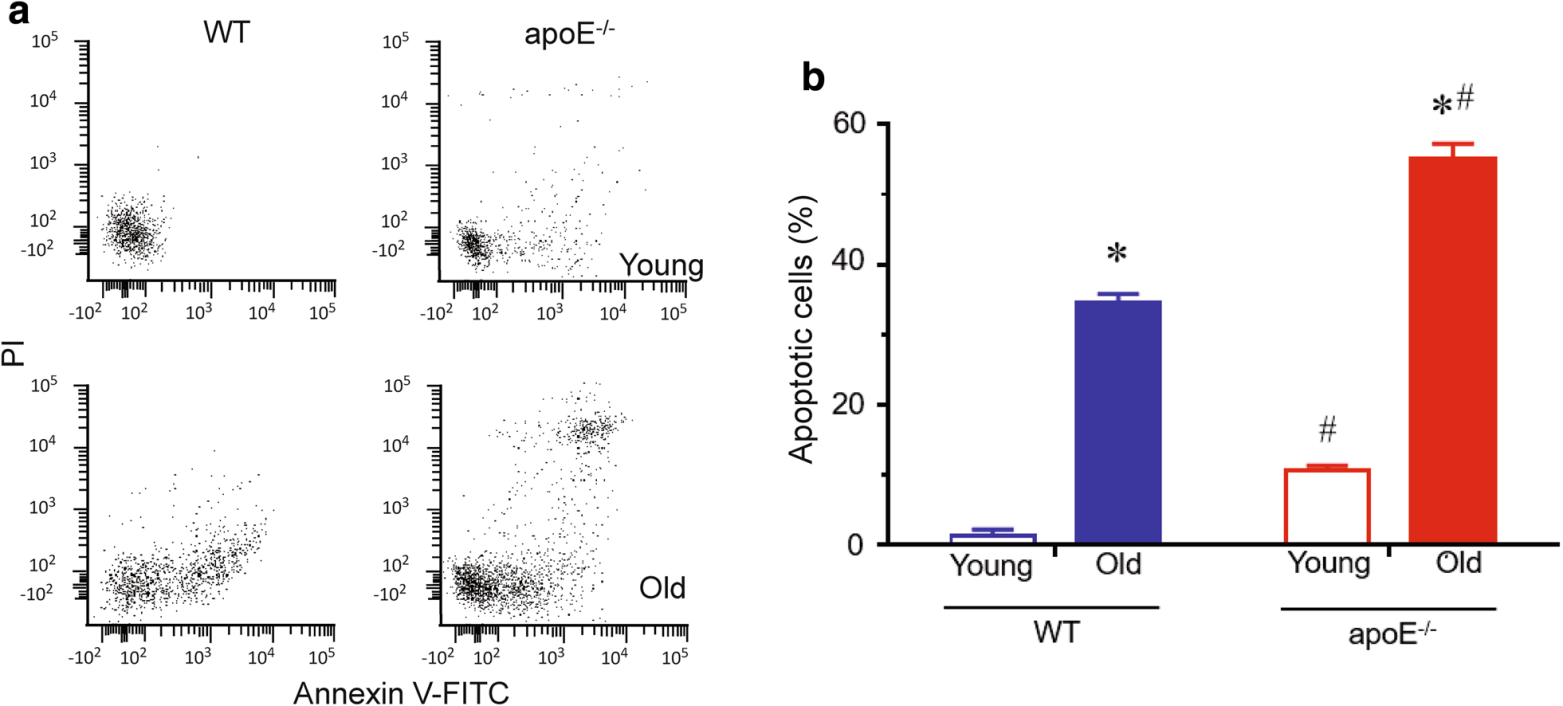
Flow cytometric determination of apoptosis. **a** Representative dot-plots of Annexin V^FITC^ and PI dual color flow cytometry for monocytes. **b** Bar graph shows average percentage of apoptotic cells (Q2 + Q4) from monocytes of WT and apoE^−/−^ mice. Values are mean ± SEM. *p < 0.05 vs. the respective young group, ^#^*p *< 0.05 vs. age-matched WT group

Considering that during apoptosis, phosphatidylserine becomes available for annexin V binding and that PI is excluded from live cells, we evaluated the apoptosis in blood cells by flow cytometry using annexin V-FITC and PI to distinguish live from apoptotic cells. Figure 6a shows representative dot plots. The apoE^−/−^ animals showed a remarkable increase in apoptotic cell number compared to age-matched WT animals. Bar graphs in Fig. 6b showed an increased number of apoptotic monocytes in apoE^−/−^ mice when compare to age-matched WT mice (young: eightfold; old: 1, sixfold, p < 0.05). In addition, aging per se increased apoptotic cell number in WT (young: 1.3 ± 0.8; old: 34.6 ± 1.1%, p < 0.01) and atherosclerotic mice (young: 10.6 ± 0.6; old: 55.1 ± 2.1%, p < 0.01).

## Discussion

The present study shows, for the first time, that hypercholesterolemia and aging leads to monocytosis, oxidative stress and, consequently, genomic instability and apoptosis in monocytes of aged apoE^−/−^ mice. We previously demonstrated in apoE^−/−^ mice that augmented ROS production occurs in arteries, liver, bone marrow and other organs during atherogenesis [15, 20–23]. Moreover, we showed that DNA damage is an event linked with ROS production [15, 21–23], and that it is greatly aggravated by aging [15, 21]. However, despite this, there is still no evidence of the relationship between increased oxidative stress and genomic instability in monocytes of apoE^−/−^ aged mice.

As expected, and corroborating previous data from our group, apoE^−/−^ mice showed higher levels of non-HDL and HDL plasma cholesterol than those of the WT group [15, 20–23]. Moreover, no differences were observed in triglyceride values between groups. The typical images of the aorta (Fig. 4) reinforce the results previously obtained by us in mice of the same age, showing a difference of at least ~ 14-fold in lipid deposition between aged apoE^−/−^ and WT mice [15, 24]. In order to exclude metabolic parameters that could be influenced by aging and, particularly, by atherosclerosis, we also measured blood glucose and OGTT and observed that the values were similar between the groups. Moreover, recent data from our laboratory demonstrated that under a normal diet, aging does not affect other biochemical profiles in the apoE^−/−^ mouse [15].

Monocytes have a key role in atherosclerotic process since they are recruited from the bloodstream to the aorta wall during atherogenesis [25]. Previous studies revealed an association risk between leukocytosis and cardiovascular diseases [26–28]. In the present study we observed an increased peripheral monocytosis in apoE^−/−^ mice and this finding is in agreement with other studies [29–32]. Previous studies suggest that hypercholesterolemia during aging may be associated with a homeostasis disruption in bone marrow [28, 33–38], leading to an increased entry of monocytes into atherosclerotic plaque [28, 39, 40]. In addition, previous reports revealed that hypercholesterolemia increased the expression of growth factors [41] and that, in this regard, apoE acts to regulate the proliferation of hematopoietic stem/progenitor cells leading to a subsequent expansion of myeloid lineage [28]. Interestingly, this “disrupting bone marrow hypothesis” was recently confirmed in experimental apoE^−/−^ mice model by us [15] and by others [40, 41]. Taken together, these data corroborate the monocytosis observed in the present study, however the detailed mechanism underlying monocytosis has not yet been fully understood.

Many studies show that the progression of atherosclerosis is orchestrated and mediated by a complex network of pro-inflammatory cytokines and their antagonism results in limitation of plaque development [42–45]. An important finding of the present study was the overproduction of serum pro-inflammatory cytokines (IL-6, TNF-α, MCP-1 and INF-γ) observed in aged mice, as well as in young experimental atherosclerotic models [46–48], leading to an amplification of the inflammatory cascade. Accordingly, previous data demonstrated that the expression of IL-6, TNFα and MCP-1 is controlled by NF-κB gene expression [49–51], a signaling pathway activated by different factors, such as hemodynamic forces and oxidized lipoproteins [45, 52]. These evidences suggest the use of pharmacological inhibitors of NF-κB as an alternative anti-atherosclerotic therapeutic strategy [53]. However, we cannot discard a possible involvement of other cytokines. In the near future, these serum pro-inflammatory biomarkers may help to predict prognosis and evaluate the response to therapy providing a relevant approach on patient care and follow-up.

Previous studies have shown that monocytes mediate inflammatory cascades and are involved in endothelial dysfunction and ROS overproduction [54–58], it is plausible to consider that monocytosis can enhance atherogenesis [59]. Cytokines contribute to the atherosclerotic disease in two phases. In the acute one, different cytokines contribute to endothelial dysfunction, expression of adhesion molecules and leukocyte diapedesis [42, 44]. In the late phase, activated leukocytes can be permanently stimulated by local cytokines, enhancing the transformation of macrophages into foam cells via scavenger receptors and cell-oxidation [17, 24, 60–62]. Cumulative data suggest that the overproduction of serum pro-inflammatory cytokines, associated with monocytosis in late phase of atherogenesis, may also contribute to the perpetuation of the inflammatory process and triggers extravascular disorders, as has been observed by us and others [12, 15].

The close relationship between inflammation and oxidative damage [6, 15, 22, 63] is supported by previous studies from our group demonstrating that virtually all cells from classical target organs of cardiovascular diseases are affected by ROS overproduction [7, 15, 20–23, 64, 65], mainly by the NADPH oxidases, which also are the main sources of ROS in phagocytic cells [66]. The present findings support the idea that the deleterious effects of ROS are not restricted to hypercholesterolemia and that they may be enhanced by aging, can occur in classical target tissue (e.g. endothelial and smooth muscle cells, and plaque macrophages) and in peripheral blood monocytes, contributing to the progression of atherosclerosis.

In the present study, we observed that the development of monocytosis in aged and in dyslipidemic mice is associated with an increased level of serum pro-inflammatory cytokines and ROS overproduction in circulating monocytes and in aorta sections. We and others have previously demonstrated that ROS overproduction may promote DNA damage through many pathways, since they are well-known genotoxins [6, 22, 23, 67–69]. The novelty of our research is the observation of an increased DNA damage in monocytes of aged and atherosclerotic animals. We demonstrated previously that ROS overproduction leads to DNA double-strand breaks and increased micronuclei formation in apoE^−/−^ mice, confirming the genotoxic, mutagenic and cytotoxic effects of hypercholesterolemia in bone marrow cells [15, 23]. Thus, in accordance with those previous data, our study also indicates that ROS production may proportionally contribute to DNA damage [6, 15, 22, 23, 70, 71].

Although it is well stablished that ROS overproduction plays a central role in cell growth, differentiation and apoptosis [6, 64, 72], the present data provides new insights by showing increased circulating apoptotic monocytes in aged mice compared to WT mice, and that this process was aggravated by hypercholesterolemia. A reasonable explanation for this finding could be that the transmigration of monocytes is mediated by pro-inflammatory cytokines and, in advanced plaques, macrophage-derived monocytes are the major contributors to oxidative stress and, consequently, to the inflammatory response [73, 74]. The macrophage-derived monocytes lipid metabolism can become overwhelmed, leading to increased lipid deposition and the growth of atherosclerotic lesions [75]. We speculate that the recruitment of apoptotic monocytes to the plaque could contribute to atherosclerosis progression by the suppression of efferocytosis [12, 76–79], culminating in the retention of lipoproteins and modification of crosstalk among cytokine signaling pathway [80]. Efferocytosis of apoptotic macrophages plays an important role in the earlier stages of atherosclerosis, as it suppresses the proinflammatory signals from foam cells [81]. During aging, this process becomes compromised, leading to an amplification of the proinflammatory signals and monocytes recruitment [82, 83]. This efferocytosis failure could be explained by the expression of CD47 antigen in the plaque cells, which impairs the recognition of apoptotic cells by efferocytes [84] and by the accumulation of oxidized LDL, which competes for apoptotic cell recognition [85].

ROS and DNA damage data suggest that the increased apoptosis of monocytes that we observed in apoE^−/−^ animals, contribute to the progression of atherosclerosis [79]. However, we cannot attribute all the changes to binomial aging and hypercholesterolemia as apoE gene itself may participate in the clearance of apoptotic cells remnants and stimulate a pro-inflammatory response [86, 87]. In addition, increased ROS production is the first signal of monocytes dysfunction, and the regulation of this process is critical in the development of atherosclerosis [88]. The upregulation of the C–C chemokine type 2 receptor (CCR2) [89, 90] and CCR5 [91] stimulates monocyte recruitment to the atherosclerotic plaques. In chronic inflammatory diseases like atherosclerosis, monocytes with macrophage-like behavior are predominant, and increase ROS production, propagating inflammation and oxidative stress [92]. Therefore, circulating monocytes could act as systemic sensors, suggesting that these cells could be used for monitoring, and even an attractive therapeutic target for the prevention or treatment of atherosclerosis.

Since all the types of cytokines were not completely evaluated, this study could possibly be a source of diagnostic bias. Last, but not least, results of this study might not be completely generalizable to other experimental models because apoE protein shows antioxidant and immune modulatory effects, as described above.

## Conclusions

In conclusion, we observed that aged mice exhibited an increased systemic pro-inflammatory response associated with an impairment of functionality of monocytes, showing an augmented oxidative stress, DNA damage and apoptosis, which was aggravated by hypercholesterolemia. Therefore, our results may contribute to a better understanding of the progression of atherosclerosis due to age-related changes in circulating monocytes. The present data open new avenues for the development of future strategies for treating atherosclerosis. Further studies are needed to elucidate alternative pathways to atherosclerotic process in this mice model.

## Methods

### Animals

Experiments were performed in 2- and 18-month-old male wild-type C57BL/6 (WT, n = 8) and apoE^−/−^ (n = 8) mice, bred and maintained in the animal care facility at the Laboratory of Translational Physiology in the Health Sciences Center at the Federal University of Espirito Santo, Brazil. The mice were housed in individual plastic cages with a controlled temperature (22–23 °C) and humidity (60%) and were exposed to a 12:12-h light–dark cycle. All mice were fed a standard chow diet and had access to water ad libitum. All experimental procedures were performed in accordance with the guidelines for the care and handling of laboratory animals as recommended by the National Institutes of Health (NIH), and study protocols were previously approved by the Institutional Animal Care Committee (CEUA-Emescam, Protocol # 014/2011).

#### Oral glucose tolerance test (OGTT)

To evaluate insulin resistance in all groups, fasted mice were administered orally with 2 g of glucose/kg body weight, and blood glucose was checked at regular intervals (0, 15, 30, 60, and 120 min) through tail blood extraction. For this purpose, the animals were placed in a restrainer and the tail section was covered with a clean gauze swab with lidocaine cream (4%) in the last 4 mm. After 2 min, the anaesthetic was removed with ethanol solution (70%) and the last 1 mm of the tip of the tail was then removed using sterilized surgical scissors. The tail was then gently massaged to ensure an adequate blood flow. The total glucose response versus time was evaluated by area under the curve (AUC) using Prism software (Prism 6.0, GraphPad Software, Inc., San Diego, CA, USA).

#### Biochemical parameters

Venous blood was collected by intracardiac puncture from the right ventricle of C57 and apoE^−/−^ mice euthanized with a sodium thiopental overdose (100 mg/kg, i.p.) to determine serum concentrations of glucose, triglycerides, total plasma cholesterol, high density lipoprotein (HDL) and non-HDL, using an automatic spectrophotometry analyzer supplied by collaborators of a clinical analysis laboratory (Tommasi Laboratory, Vitoria, ES, Brazil). Non-HDL levels were calculated by subtracting HDL from total serum cholesterol.

### Isolation of blood cells and flow cytometry

Blood samples collected were mixed with lysing Buffer 1× (Amersham Biosciences, Piscataway, NJ) for 5 min at 37 °C to remove erythrocyte. Cell suspension was centrifuged for 10 min at 1200 rpm, supernatant was discarded and the pellet washed in phosphate-buffered saline (PBS) plus 10% Fetal Bovine Serum (FBS) for 10 min at 1200 rpm. Cells were collected and resuspended in 1 mL PBS with 10% FBS. Erythrocyte-depleted intact blood cells were counted and analyzed by flow cytometry. For specific leukocytes population analysis, in a cell population scatter plot displaying the FSC (x-axis) and SSC (y-axis) of lysed whole blood graph, monocytes were gated for flow cytometry analysis as shown in Fig. 1a. The different physical properties of monocytes allow them to be distinguished from cellular contaminants. For the confirmation of monocytes population among whole blood cells, the samples were incubated with allophycocyanin (APC)-labeled anti-mouse CD11b (BD: Becton–Dickinson Biosciences, San Jose, CA, USA) (Fig. 1b). After blood collection, the atherosclerotic-associated macroscopic changes in aortic arch were visually determined (Fig. 4a).

#### Macroscopic and microscopic evaluation of aortic arch plaque

For the macroscopic evaluation, after blood collection the animals were perfused, the aorta was delicately isolated, and the connective tissue was removed to allow visualization of any the atherosclerotic-associated changes in the aortic arch, particularly plaque deposition (Fig. 4a).

In addition, for microscopic visualization of the aortic arch, mice were euthanized (sodium thiopental, 100 mg/Kg, IP) and perfused via the left ventricle with phosphate–buffered saline (PBS, pH 7.4; 01 M) followed by a fixative solution of formaldehyde (4%). The aortic arch was dissected, removing removing the connective tissue. After resting overnight in the fixative solution, the aortic arch were embedded in methyl methacrylate resin and 5 μm sections were prepared. The samples were stained with hematoxylin and eosin, and images were captured with a video camera (AxioCam ERc5s, Carl Zeiss, Germany) with 40× magnification.

### Analyses of cytokines with flow cytometric bead-based assay

The cytometric bead array (CBA) mouse inflammation kit (BD) employs a series of particles with discrete fluorescence intensities to simultaneously detect multiple soluble analytes, which were measured in particle-based immunoassay through the sensitivity of amplified fluorescence detection by flow cytometry. In the CBA-Mouse Inflammation Kit (CBA-Mouse) system from BD, six bead populations with distinct fluorescence intensities are coated with capture antibodies specific for IL-6, IL-10, MCP-1, INF-ɤ, TNF, and IL-12p70 proteins [6]. The six bead populations are mixed together to form the BD CBA which is resolved in a red channel of a flow cytometry. The capture beads, PE-conjugated detection antibodies, and recombinant standards or test samples are incubated together to form sandwich complexes. Cytokines levels were measured in plasma, according to the manufacturer’s instructions (BD). For these analyses, a typical forward and side scatter gate was set to exclude aggregates; a total of 5000 events in the gate were analyzed using FACSCanto II and FACSDiva Software (BD). Samples were quantified by comparison with standard curves of recombinant mice cytokines using FCAP Array software. The results were expressed as pg/mL.

### Measurement of intracellular reactive oxygen species

The ROS analysis was performed by flow cytometry as previously described [6, 15, 20, 21]. Given its ability to freely permeate cell membranes, dihydroethidium (DHE) has extensively been used to monitor ROS production. To estimate the ROS production, approximately 100 µL of erythrocyte-depleted intact blood cells were incubated with 20 µL of DHE (160 µM) for 30 min at 37 °C in the dark to load the cells with the dye. For positive control, samples were treated with 10 μM doxorubicin for 5 min to create oxidative stress without cell toxicity. Cells incubated with ethanol were used as the negative control. Cells were then washed, resuspended in PBS, and maintained on ice for immediate acquisition by flow cytometry (BD). Data were analyzed using the FACSDiva software (BD), and overlay histograms were constructed using the FCS Express software. For fluorescence quantification, samples were acquired in duplicate, and 10,000 events were registered from each sample. Intensity of specific fluorescence was expressed as the median fluorescence intensity (MFI), from the average of at least three repeated experiments (a.u.).

In addition, to detect ROS production in aorta, unfixed frozen sections from the artery were cut in 7-µm-thick sections and mounted on gelatin coated glass slides. Samples were incubated with DHE (2 µmol/L) in a modified Krebs’s solution (containing 20 mM HEPES), in a light-protected humidified chamber at 37 °C for 30 min [22]. The fluorescence intensity was quantified by a blind investigator using a fluorescence microscope (Nikon Eclipse TI, Nikon Instruments Inc., Melville, NY, USA). Data is expressed in arbitrary units.

### DNA content analysis

Cell cycle distribution was determined by flow cytometry analysis of the DNA content. Briefly, erythrocyte-depleted intact blood cells were incubated with 200 µL of staining solution (20 mg/mL RNAse A, 500 mg/mL propidium iodide [PI], 1% Triton X-100) for 30 min at 4 °C, in the dark. Then, cells were washed, centrifuged for 10 min at 1200 rpm, and resuspended in PBS. Samples were acquired in triplicate and 10,000 events were used for each measurement. The cell cycle profile was determined using a FACSCanto II flow cytometer. For determination of DNA content, samples were acquired in triplicate and 10,000 events were used for each measurement. The fragmented low-molecular weight DNA can be observed to the left of interphasic DNA (2n) cells peak [20]. Data analysis was performed by the FACSDiva software.

### Apoptosis

Apoptotic cells were determined by the loss of plasma membrane integrity, which was characterized by the translocation of the phospholipid phosphatidylserine from the inner to the outer leaflet allowing the binding of the annexin V protein to cells with exposed phosphatidylserine. 200 µL erythrocyte-depleted intact blood cells were resuspended in binding buffer 1× (10 mmol HEPES, NaOH, pH 7.4, 140 mmol NaCl, 2.5 mmol CaCl_2_). Then, 100 µL of this solution were transferred to a new tube and incubated with 5 mL of Annexin V-FITC and 5 mL of PI for 15 min at room temperature (25 °C) in the dark, using a commercial kit according to the manufacturer’s instructions (BD). Finally, 400 μL of 1× binding buffer was added to each tube and the samples analyzed by a FACSCanto II flow cytometer (BD) within 1 h. All data analyses were performed using FACSDiva analysis software (BD). For quantification of apoptotic cells, samples were acquired in triplicate and 10,000 events were used for each measurement and data expressed as the percentage of positive cells. The percentage of cells was determined in Q_1_ (AnnexinV^FITC−/PI+^, damaged cells), Q_2_ (Annexin V^FITC+/PI+^, end stage apoptotic cells), Q_3_ (AnnexinV^FITC−/PI−^, viable cells), and Q_4_ (Annexin V^FITC+/PI−^, early apoptotic cells) [6, 15, 20, 21]. The apoptotic rate of cells undergoing apoptosis was determined as the percentage of Q_2_ + Q_4_.

### Statistical analysis

All data are expressed as mean ± SEM. The normality (Gaussian distribution) of the variables was previously analyzed using the Kolmogorov–Smirnov test. When this test was significant, the statistical analysis was performed using two-way analysis of variance (ANOVA) followed by Tukey’s post hoc test. The analyses were performed using Prism software (Prism 7, GraphPad Software, Inc., San Diego, CA, USA). The differences between means were considered significant when p < 0.05.

## Abbreviations

apoE^−/−^: apolipoprotein E knockout mice
CBS: cytometric bead-array
DHE: dihydroethidium
FACS: fluorescence activated cell sorting
FBS: fetal bovine serum
FITC: fluorescein isothiocyanate
FSC: forward scatter
IL: interleukine
INF: interferon
i.p.: intraperitoneal
MCP: monocyte chemoattractant protein
MFI: median fluorescence intensity
NF-κB: nuclear factor kappa b
PBS: phosphate-buffered saline
PI: propidium iodide
ROS: reactive oxygen species
SSC: side scatter
TNF: tumor necrosis factor
WT: wild type
